# The potential of PLA based dental models by material extrusion 3D printing: an in vitro study investigating mechanical properties and dimensional accuracy

**DOI:** 10.1007/s10856-025-06899-y

**Published:** 2025-06-10

**Authors:** Jiandong Li, Yuyang Mao, Jamila Yassine, Nico Henning, Alexey Unkovskiy, Florian Beuer, Franziska Schmidt

**Affiliations:** 1 Charité - Universitätsmedizin Berlin, Corporate Member of Freie Universität Berlin, Humboldt-Universität zu Berlin, and Berlin Institute of Health, Dental Materials and Biomaterial Research, Department of Prosthodontics, Geriatric Dentistry and Craniomandibular Disorders, Aßmannshauser Str. 4-6, 14197 Berlin, Germany; 2 Charité - Universitätsmedizin Berlin, Corporate Member of Freie Universität Berlin, Humboldt-Universität zu Berlin, and Berlin Institute of Health, Department of Operative, Preventive and Pediatric Dentistry, Aßmannshauser Str. 4-6, 14197 Berlin, Germany; 3 Technische Universität Berlin, Faculty III - Process Sciences, Institute of Materials Science and Technology, Chair of Materials Science & Engineering/Fachgebiet Werkstofftechnik, Str. des 17. Juni 135, Berlin, 10623 Germany; 4 Department of Dental Surgery, Sechenov First Moscow State Medical University, Bolshaya Pirogovskaya Street, 19с1, Moscow, 119146 Russia

## Abstract

The rapid advancement of three-dimensional (3D) printing in dentistry has prompted comparisons between the mechanical properties of polylactic acid (PLA) samples fabricated using material extrusion (MEx) and resin samples produced using digital light processing (DLP). This study aims to assess the potential of replacing resin-based models with PLA models in clinical settings by evaluating the mechanical properties and accuracy of MEx-printed PLA and DLP-printed resin samples. The investigated materials include pure PLA, a PLA composite containing gypsum, and a clinically approved resin material. Strength and hardness tests were conducted using custom-made samples measuring 16 × 4 × 2 mm^3^. Additionally, oral cavity scans were used to generate oral models for each material to assess their accuracy, trueness, and precision. The results indicated that pure PLA exhibited the highest flexural modulus (2055 ± 217.70 MPa) and compression modulus (2.40 ± 0.14 GPa). The PLA-Gypsum composite displayed the highest hardness (19.48 ± 2.12 HV1). As for the trueness of the oral models, there were no statistically significant differences between the models made from the three materials. However, the PLA-Gypsum composite demonstrated the best precision (23.84 ± 4.12 μm). These findings suggest that both PLA materials have significant potential to replace DLP-produced resin models in the clinical applications.

**Graphical Abstract:**

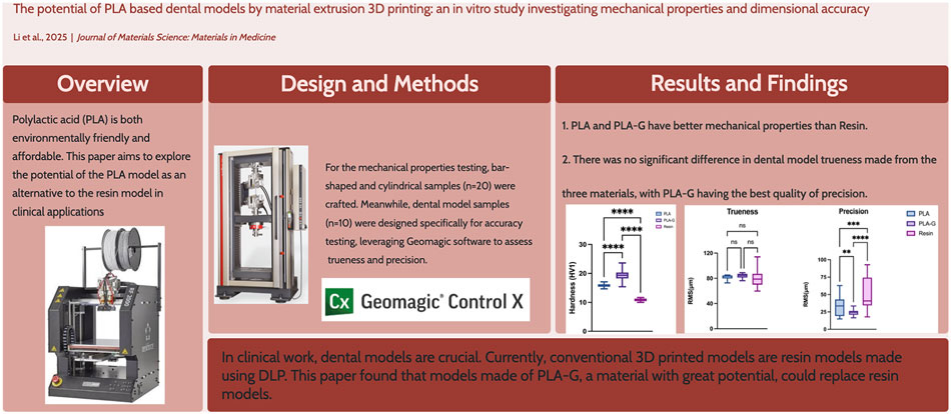

## Introduction

In additive manufacturing (AM), commonly known as three-dimensional printing (3D printing), materials are deposited layer by layer to create a physical objects based on digital models. The process involves using computer-aided design (CAD) and computer-aided manufacturing (CAM) technologies, along with imaging and scanning systems, to create the desired structure or model [1]. AM technology has been used in industrial manufacturing for several decades, however the high cost of some 3D printers and materials restricts their potential for broader application [2]. In spite of this, its personalized and precise nature has made it increasingly popular in various fields, including in clinical treatments [3]. AM has been employed in medical and dental applications to produce tissues, organs, and prostheses for surgical operations as well as accurate models, retainers, aligners, and oral implant guides[4]. The technology is particularly useful in dentistry, where the most common AM technologies and devices for polymers and polymer-based composites are resin-based methods of Vat-Photopolymerization, in specific stereolithography (SLA) and digital light processing (DLP), as well as powder bed fusion methods for metals processing [5].

In Fused filament fabrication (FFF), also known as Fused Deposition Modeling (FDM), a thermoplastic filament is heated and then extruded through a nozzle to produce three-dimensional structures [6]. FFF/FDM is classified in the standard ASTM F2792 - 12a as a material extrusion technology [7]. The MEx technology has great popularity due to low prizes and accessible materials and has gained wide acceptance over the past decade, showing the potential to revolutionize manufacturing across a wide range of industries [8]. Several researchers have been attracted to MEx printers because of their affordability and user-friendliness, as well as the minimal possibility of material contamination [9]. The properties of PLA make it an excellent alternative to conventional petrochemical-based polymers in various industrial applications since it is biocompatible, eco-friendly, biodegradable, and renewable. With its inherent versatility and eco-friendliness, PLA is a highly attractive material for a wide range of applications, including consumer products as well as medical implants As soon as PLA is injected into living organisms, including the human body, it undergoes hydrolysis, breaking down into the hydroxy acid constituent, which is subsequently incorporated into the tricarboxylic acid cycle and excreted from the body [10]. The application of polylactic acid (PLA) in dentistry has gained attention in recent research. Hamed et al. [11] applied PLA to fabricate temporary crowns, assessing factors such as discomfort, breakage, and movement of these crowns. Tyler et al. recently demonstrated that PLA can enhance successful osseointegration of dental implants with surrounding native oral hard tissue [12]. Additionally, Ranjbar et al. [13] incorporated PLA composite into resin formulations, resulting in improved mechanical properties compared to conventional resins. It should be noted, however, as a solution to weak mechanical properties of PLA, researchers have investigated the use of additives to enhance the properties. A number of natural fibers have been extensively studied for their ability to improve the mechanical properties of PLA, including wood, hemp, kenaf, bamboo, sugarcane bagasse, and flax [14]. It is noteworthy that, despite the use of the same additives, PLA composites can differ in their mechanical strength depending on factors such as the proportions of additives and the conditions under which they are manufactured [15]. As a result, selecting additives carefully and optimizing printing conditions is critical for achieving desired properties when 3D printing PLA. It is clear that the incorporation of additives into pure PLA offers a potential approach for improving the properties of PLA-based materials while overcoming the weaknesses associated with MEx printing [16, 17]. Research has shown that gypsum powder is well-suited for 3D printing, resulting in samples with a better performance of complex structures [18]. In their research, Stoof and colleagues demonstrated that incorporating a specific proportion of gypsum into the filament utilized for 3D printing diminishes the shrinkage observed in the fabricated samples [19].

In dentistry, especially orthodontics, digital light processing (DLP) and stereolithography (SLA), both vat photopolymerization techniques (according to the ASTM F2792) [7] have been widely used in clinical applications [20]. DLP printers employ a light source that is controlled by a digital micro-mirror to polymerize a photosensitive liquid resin. This technique is applicable to various resin systems and has been demonstrated to produce models with a high degree of accuracy [21]. However, DLP printing technology isn’t without its drawbacks. Compared to MEx technology, the cost of DLP printers and the materials used for printing is significantly higher. Furthermore, DLP technology is limited to producing smaller models. Additionally, the performance of photocured materials is a concern; they are brittle, prone to deformation, more post-treatment compare to MEx printer and have bad weather resistance [22]. All these factors suggest that MEx 3D printing technology holds great potential to overtake DLP technology in model production. Despite the widespread application of MEX 3D printers for PLA modeling, there remains a notable gap in the literature concerning dental models. Particularly, there is a lack of systematic comparison involving the mechanical properties of PLA, PLA composites, and resin, including aspects such as model accuracy, density, and surface roughness. This study provides an in-depth discussion of the above critical issues.

Dental models play a critical role in various dental applications, and the precision of these models significantly influences clinical outcomes. Traditionally, the creation of dental models has involved the manual process of taking impressions, a method heavily reliant on the expertise and experience of dental professionals [23]. However, with the advent of Computer-Aided Design and Computer-Aided Manufacturing (CAD/CAM) technology, the conventional approach to model fabrication has seen a shift [24]. Through the utilization of oral scanners, it is now possible to perform direct scanning of the patient’s teeth and soft tissue, enabling the direct 3D modeling of the patient’s intraoral condition, this process, known as oral digital modeling, obviates the need for physical storage space, as models can be digitally saved and easily accessed for printing via 3D printers when necessary [25]. It is important to note that when using a 3D printer, it is essential not only to set the correct parameters but also to consider the print orientation of the sample. Experiments conducted by Unkovskiy et al. [26] and Metin et al. [27] have demonstrated that print orientation significantly impacts both the accuracy and the mechanical properties of the printed sample. Despite the wide application of Digital Light Processing (DLP) printers in dentistry, the potential benefits of using MEX 3D printing technology to create Polylactic Acid (PLA) dental models should not be overlooked. This study aims to investigate whether dental models produced using MEx technology can serve as viable alternatives to those fabricated with DLP technology, with a specific focus on model accuracy.

The aim of this article is to conduct a comprehensive examination of the mechanical properties of two AM methods with three distinct materials, namely for MEX a commercial PLA filament and a PLA-Gypsum composite filament, and for DLP a resin for producing dental models as the control group while testing the trueness and precision of dental models of these three different materials produced by AM. This study establishes a theoretical foundation for the clinical use of dental models made from PLA, possibly contributing to reduced hospital expenses and reducing environmental damage. The null hypothesis posits that there are no significant differences in the mechanical properties and the accuracy (trueness and precision) of the three materials. This study intends to provide valuable insights into the material selection for suitable applications in dentistry.

## Materials and methods

### Model design and sample preparation

The research involved the utilization of three distinct materials, namely PLA (batch number:78691362020, 3Dmensionals, Freiburg, Germany), PLA-Gypsum composite (Filadental Gips, reference: 4251169217099, 3dk.berlin, Berlin, Germany) (abbreviation: PLA-G), and Resin (Luxaprint Model BGE, lot: 284077, DMG, New Jersey, USA), to generate samples in three varying shapes: bars, cylinder, and dental models (DM). A total of 20 samples of both bar (*n* = 20 per group) and cylinder shape (*n* = 20 per group) were produced for each material, while 10 samples of the dental model (*n* = 10 per group) were created. In addition, a set of five small bar samples per material was produced to allow an evaluation of the hardness properties of each material.

The CAD (Computer Aided Design) model of a small bar (2 × 4 × 16 mm³) and cylinder (diameter 6 mm, height 12 mm) was created using Blender (version 2021, Blender Foundation, Amsterdam, Netherland) resulting in an STL (Surface Tessellation Language) file. The CAD (Computer Aided Design) model of the dental model (DM-CAD) was scanned from a Charité Medical University Berlin patient as an STL file.

### 3D printing

The commercial PLA and PLA-G filaments with a diameter of 1.75 mm were processed in an MEx 3D printer (RF2000v2, Conrad Electronic SE, Hirschau, Germany). Printing parameters and print strategy were accomplished with the software CURA Version 5.3 (Ultimaker, Utrecht, Netherlands).

The printing parameters of models are shown in Table 1. The filament samples were printed to achieve optimum properties, so according to the manufacturers suggestions variations in nozzle temperature and printing speed may occur.

**Table 1:** The printing parameters of PLA and PLA-G samples

	Printing Speed (mm/s)	nozzle temperature (°C)	Layer height (mm)	Layer width (mm)	Infill pattern	Nozzle diameter (mm)	Bed temperature (°C)
PLA							
Bar	5	215	0.2	0.4	Concentric	0.4	60
Cylinder	5	215	0.2	0.4	Concentric	0.4	60
Dental model	15	215	0.2	0.4	Triangle	0.4	60
PLA Gypsum							
Bar	5	205	0.2	0.4	Concentric	0.4	60
Cylinder	5	205	0.2	0.4	Concentric	0.4	60
Dental model	15	215	0.2	0.4	Triangle	0.4	60

For the resin material a DLP 3D printer (3Demax, DMG, New Jersey, USA) was employed. The printing parameters were set by the software Autodesk Netfabb Standard 2022 (Autodesk, San Francisco, California, USA).

**Fig. 1:**
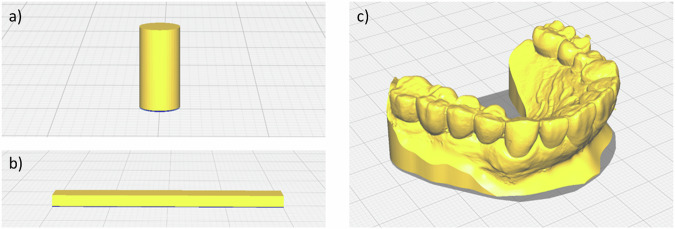
Printing design and direction in Mex for each sample geometry: **a** cylinder for compression, **b** bending bars, **c** dental model

To ensure optimal results, the resin samples were printed using the default program (LP Model, layer size: 100 μm) supplied by the manufacturer DMG for the nesting software Autodesk Netfabb Standard 2022. The samples were positioned flat at the platform (Fig. 1). After printing, the samples were ultrasonically cleaned using the state-of-the-art DMG attached machine 3Dewash with isopropanol for a total of 330 seconds with the automatic program suggested by the manufacturer. Lastly, the samples were post-cured in the 3Decure equipment (both DMG, New Jersey, USA) following the program suggested by the supplier for the DMG LuxaPrint Model BGE program, a total of 7 min.

### Analysis methods

An optical microscope VHX-5000F (Keyence, Osaka, Japan) was used to analyze the surface structure of the samples. Density measurements were performed with an Archimedes density measuring instrument (KERN YDB-03, KERN & SOHN GmbH, Balingen, Germany); the porosity of the samples can be determined using the following equation (1):

$${\rm{Porosity}}=1-({\rm{sample}}\; {\rm{density}}/{\rm{filament}}\; {\rm{density}})\times 100 \%$$

resin original material is liquid is not included in the calculation. For pure PLA filament the density was taken from the material data sheet. The density of PLA-G filament was measured by Archimedes as explained above, to estimate the porosity of the printed. To do this, 10 pieces of filament were prepared, each approximately 1.5 cm in length, to measure their density and establish the theoretical density. Digital calipers were used to measure the geometry of the bars and cylinders. A universal testing machine (Z010, ZwickRoell GmbH & Co, Ulm, Germany) was used to perform three-point bending and compression tests at a constant speed of 1 mm/s and a preload of 1 N. The hardness test was performed on a micro hardness tester named Qness Q10M (QATM, Hassloch, Germany); HV1 (Vickers Hardness with 1 kg of load) was determined; each sample was tested in five different positions, and five specimens were tested per group. A Blue LED Multi-line scanner (D2000, resolution: 5μm, 3shape, Copenhagen, Denmark) was selected as the reference scanner to scan the DM for each group. The data of trueness (trueness can be defined as the deviation between the printed object and the actual object’s dimensions, here we compared the surface scan of each DM with the CAD file of the initial DM) and precision (precision is defined as the deviation between repeated prints on the same object) [28, 29] of DM were performed using the Geomagic Control X software (3D Systems, Rock Hill, USA). DM models made from three different materials were scanned using the D2000 scanner and exported as STL files. The DM samples from each material were labeled numerically as No. 1-10. For trueness test, the CAD file for the DM was uploaded to Geomagic Control X software, using it as a standard model. To enhance the accuracy of the superimposition, irrelevant areas beyond the designated region of interest were excluded, while the region of interest include teeth, gums, and hard palate. After uploading the surface scans of the DM models into Geomagic Control X, an Initial alignment was performed. Following this, a Best Fit Alignment was executed after selecting the region of interest again. A 3D matching was then conducted with the same region of interest selected with the specific tolerances set to ±20 μm. For each material group 10 measurements were performed, the RMS (Root Mean Square) values and color-code map from each measurement were exported as a result. For the precision test, sample No.1 was first uploaded as the standard model, and the same region of interest was selected. Then, DM samples No. 2 through 10 were individually uploaded for testing to obtain their respective RMS values. Next, DM model No. 2 was uploaded as the new standard model, with DM samples No. 3 through 10 uploaded separately for further testing to derive RMS values. This process was repeated, resulting in a total of 45 RMS values for each material. The samples distributions were tested for normality, the data of trueness and precision were not normally distributed, therefor the hypotheses were tested using non-parametric tests. Prism 9.0 (GraphPad, Boston, MA, USA) was used to perform statistical analyses of variance (ANOVA) to identify significant differences and draw graphs of the results. The level of significance was set at *p* < 0.05. In the statistical figure different symbols were used to show results, “ns” (not significant) denotes *P*-value ≥ 0.05, “*“ denotes 0.01 < *P*-value < 0.05, “**“ denotes 0.001 < *P*-value < 0.01, “***“ denotes 0.0001 < *P*-value < 0.001, “****“ denotes *P*-value < 0.0001.

## Results

### Density and porosity rate

The porosity and density of printed PLA and PLA-G samples are shown in Table 2. The porosity of PLA-G is higher than that of pure PLA, regardless of the geometry (bar or cylinder).

**Table 2:** Density of the printed bars and cylinders, and the filament density of original materials

	Pure PLA	PLA-G	Resin
Density (g/cm³)			
Bar	1.23 ± 0.01	1.29 ± 0.01	1.22 ± 0.01
Cylinder	1.20 ± 0.01	1.25 ± 0.03	1.22 ± 0.01
Filament density	1.25^a^	1.33 ± 0.01	n/a
Calculated porosity (%)			
Bar	1.6	3.0	n/a
Cylinder	4.0	6.0	n/a

Data is given as mean ± standard deviation (*n* = 10/group).

^a^Density data for the pure PLA filament is derived from the material data sheet

### Mechanical properties

#### Bending and compression test

**Fig. 2:**
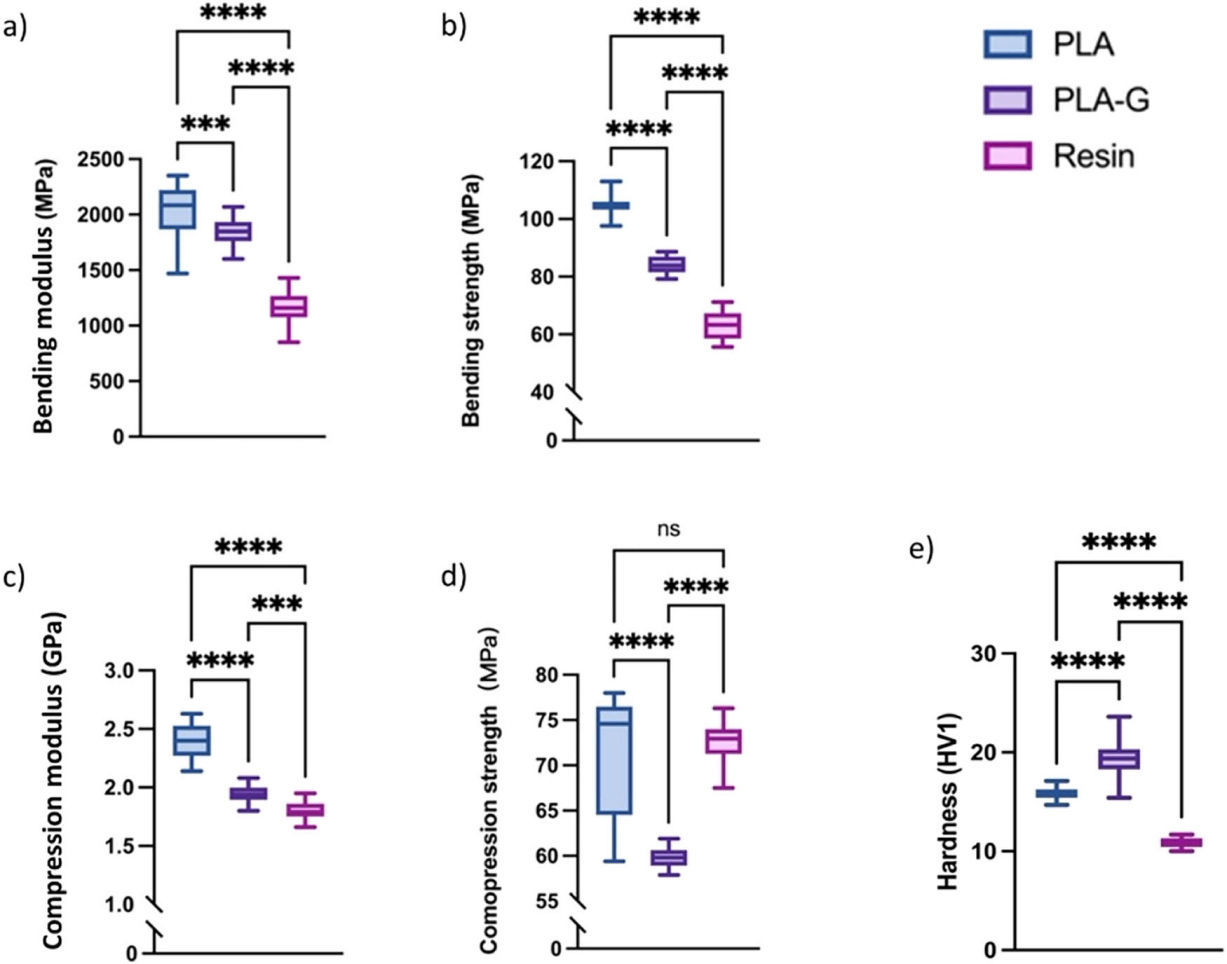
Box-plots of the mechanical characterization of the three materials: **a** bending modulus, **b** bending strength, **c** compression modulus, **d** compression strength and **e** Vickers hardness. In the figure, “***“ denotes 0.0001 < *P*-value < 0.001, “****“ denotes *P*-value < 0.0001, “ns” denotes *p*-values > 0.05

The results of bending modulus, bending strength, compression modulus and compression strength from three-point bending tests conducted on small bar-shaped specimens and compression tests on cylinder shaped samples are presented in Table 3, while the statistical data for the tests per group is illustrated in Fig. 2a, b) for bending, Fig. 2c, d) for compression. The statistical analysis indicated that all three materials showed significant differences in bending modulus and bending strength when compared to each other. For compression test all three materials showed significant differences in compression modulus, while PLA and PLA-G, as well as PLA-G and resin material showed significant differences in compression strength, but PLA and resin material showed no significant differences in compression strength.

**Table 3:** The data of the mechanical properties test

	PLA	PLA-G	Resin
Bending modulus (MPa)	2.05 ± 0.22	1.84 ± 0.13	1.15 ± 0.13
Bending strength (MPa)	104.80 ± 3.46	84.01 ± 0.66	62.99 ± 4.70
Compression module (GPa)	2.40 ± 0.14	1.95 ± 0.08	1.80 ± 0.08
Compression strength (MPa)	71.61 ± 6.67	59.79 ± 1.05	72.65 ± 2.17
Hardness (Hv1)	15.88 ± 0.64	19.48 ± 2.12	10.84 ± 0.50

Data is given in mean ± standard deviation (*n* = 20).

#### Hardness test

The result of the hardness test is shown in Table 3, while the statistical results of the hardness test are shown in Fig. 2e). The statistical analysis indicated that all three materials showed significant differences when compared to each other.

### Accuracy test

**Fig. 3:**
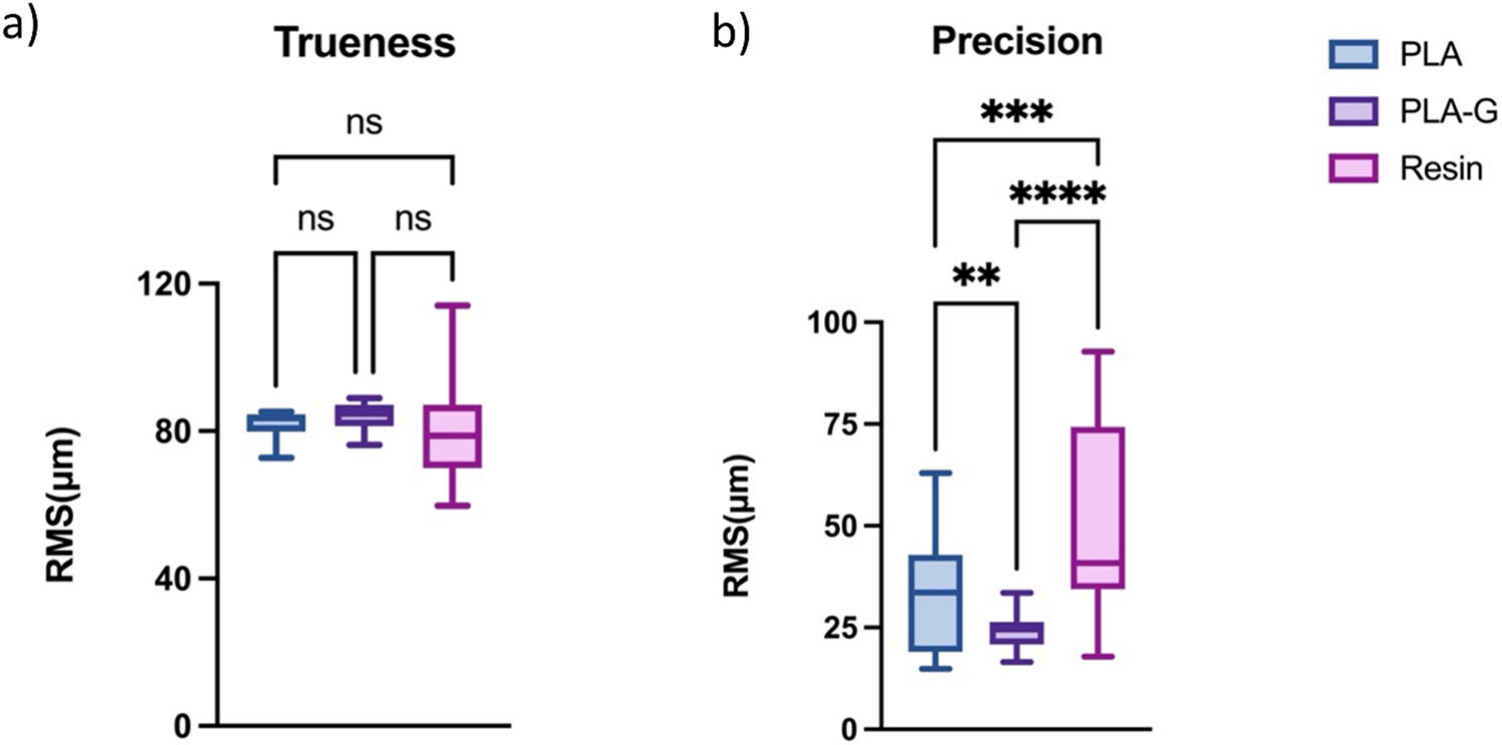
Statistical outcome and Box-plot of **a** trueness and **b** precision. In the figure, “ns” denotes *P*-value ≥ 0.05 (not significant), **denotes 0.001 < *P*-value < 0.01, ***denotes 0.0001 < *P*-value < 0.001, ****denotes *P*-value < 0.0001

**Fig. 4:**
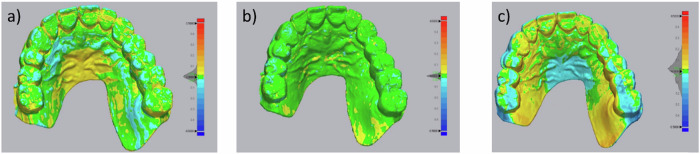
Exemplary color-coded maps (precision) of **a** PLA, **b** PLA-G, and **c** Resin samples, yellow-red color code indicates positive deviations from the DM-CAD model; light-dark blue indicates negative deviations from the DM-CAD standard model. The dark area on the bar shows the distribution of the deviation

The software Geomagic Control X was used to evaluate the trueness and precision of the printed DM samples by calculating Root Mean Square (RMS) values. After comparing the trueness measurements of different materials, no significant differences were found; there were significant differences in the mean RMS values of precision of models per material, the RMS value shown are in Table 4, and the statistical is shown in Fig. 3. The statistical analysis of trueness shows no significant differences between the RMS values of the materials, while the statistical analysis of precision shows significant differences between the materials. An exemplary color-code map of precision for each material group is shown in Fig. 4 (green surfaces present the deviations ranging between 0 and ±20 μm). The precision result of PLA-G is the most accurate.

**Table 4:** Trueness and precision values (μm) per group

	PLA	PLA-G	Resin
Trueness (μm)	81.90 ± 3.89	84.04 ± 4.22	81.13 ± 16.32
Precision (μm)	33.04 ± 13.30	23.84 ± 4.12	50.74 ± 22.71

### Simple linear regression analysis for mechanical properties and density

Based on simple linear regression analysis, it was found that the density had no statistically significant effect on bending modules for all three materials, *p* > 0.05. For PLA-G and resin material, the effect of density on compression modulus was not statistically significant; on the contrary, the density of PLA affected the compression modulus had a statistical significance value of *p* < 0.05, R^2^ = 0.3883.

### DM surface

**Fig. 5:**
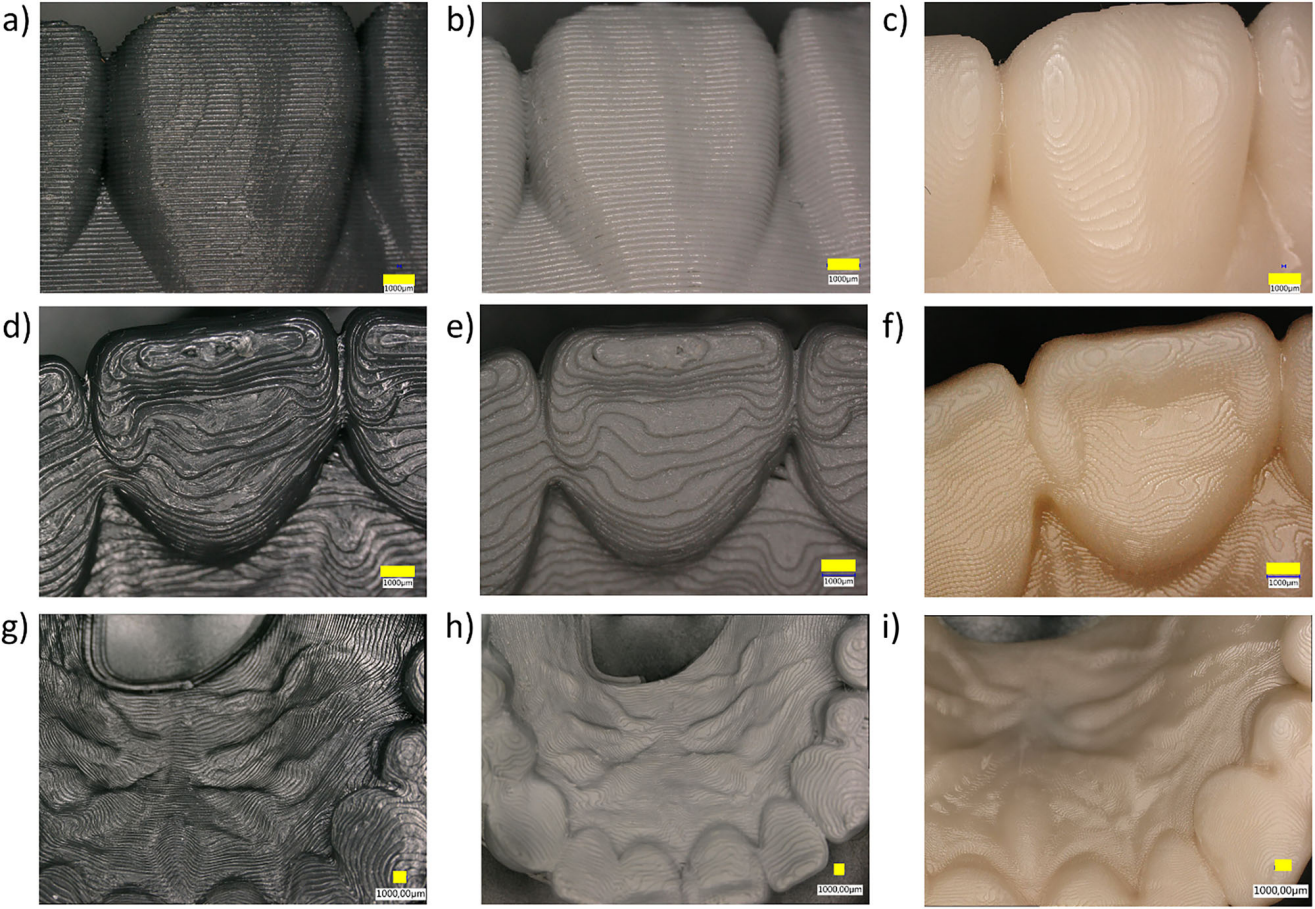
Microscope images of DM from each material, **a** buccal side of tooth #21 (PLA), x30, **b** buccal side of tooth #21 (PLA-G), x30, **c** buccal side of tooth #21 (Resin), x30, **d** lingual side of tooth #21 (PLA), x30, **e** lingual side of tooth #21(PLA-G), x30, **f** lingual side of tooth #21 (Resin), x30, **g** hard palatal side of DM (PLA), x20, **h** hard palatal side of DM (PLA-G), x20, **i** hard palatal side of DM (Resin), x20. Yellow bar on each figure indicates 1 mm length

The surface structures of the palatal side of the DM-CAD samples are shown in Fig. 5. The samples printed with resin consisted of the thinnest contour, and the smoothest surface. All materials showed visible layer structures on the surfaces.

## Discussion

The aim of this research is to compare the mechanical properties of a commercial non-dental PLA filament with a commercial dental gypsum filled PLA filament (PLA-G), and a commercial dental resin material regarding its application for producing dental models. At the same time, the comparison of the accuracy of specimen manufactured from each material by 3D printing explores the potential of using PLA or PLA-G by MEx as a replacement for resin-based additive manufacturing to produce clinical dental models. The findings of this study can provide valuable insights into the feasibility of using PLA based filaments in the MEx technology to produce high-quality clinical models. Compared to resin materials, PLA has the undeniable advantage of being cheaper, no matter the material or the 3D printer, and therefor the potential of greatly reducing healthcare costs [12]. At the same time, PLA is also a biodegradable material, and the influence of PLA medical waste on the environment is much smaller than the impact of resin [10]. In this study, we successfully produced MEx-printed PLA and PLA-gypsum composite samples, which outperformed DLP-printed resin in terms of mechanical properties and accuracy.

The application of 3D printing technology has promising potential in the field of dentistry. However, it is essential to evaluate the accuracy of 3D printed dental models to ensure their effectiveness. To the best of the author’s knowledge, this is the first time a study was conducted to assess the mechanical properties and clinical model accuracy of dental models made of three different materials- PLA, PLA-G, and resin using 3D printing technology. This study is a significant step towards understanding the potential of 3D printing technology in dentistry and improving its accuracy and efficiency.

**Fig. 6:**
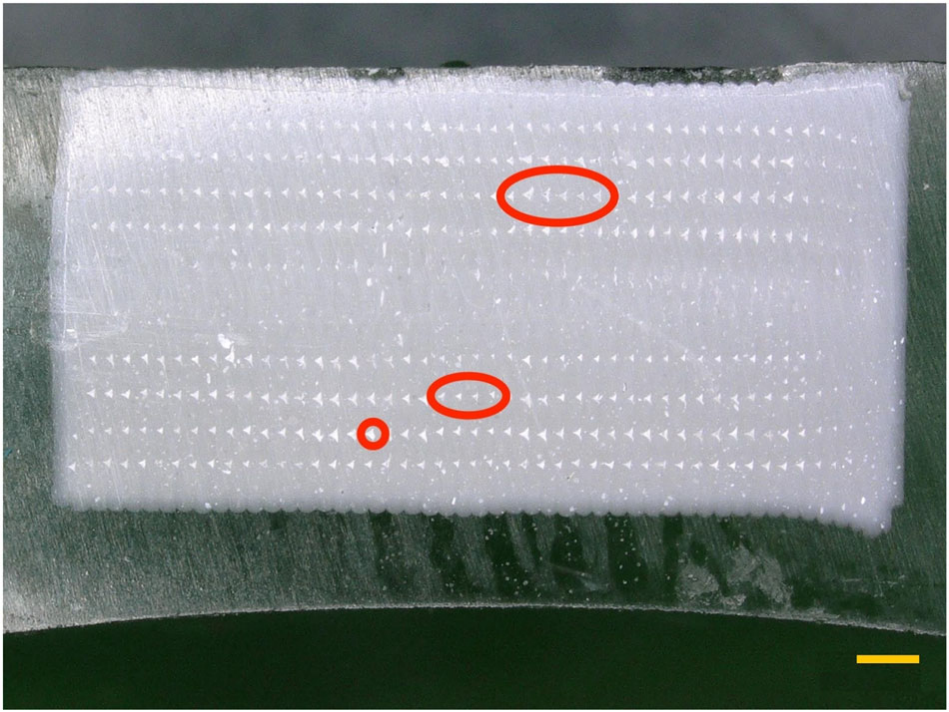
Microscopy image of a longitudinal cross section of a PLA-G cylinder sample, 30x magnification. The regions highlighted with red circles demonstrate porosity. The yellow bar on the figure indicates 1 mm length

Pure PLA materials fabricated by 3D printing show some limitations, e.g. regarding mechanical, thermal or functional properties, therefor different PLA based composites have been developed and investigated for many years. In our study unfilled PLA has demonstrated superior performance in both compression and bending tests, exhibiting compression and bending strength of 71.61 ± 6.67 MPa and 104.8 ± 3.46 MPa, respectively. PLA-G, on the other hand, exhibited compression and bending strength of 59.79 ± 1.05 MPa and 84.01 ± 0.66 MPa, respectively. PLA-G, on the other hand, exhibited compression and bending strength of 59.79 ± 1.05 MPa and 84.01 ± 0.66 MPa, respectively. The primary mechanical property utilized for evaluating the strength of clinical plaster products is compressive strength. In clinical applications, the compressive strength of plaster models typically ranges from approximately 18–38 MPa, contingent upon the specific material used [30]. Notably, the mechanical properties of all three materials tested in this experiment exhibited considerably higher strength levels than various gypsum materials commonly employed in clinical settings. The model made with PLA demonstrated the stronger resistance to external forces, owing to its higher mechanical properties as compared to the gypsum material. Therefore, it is less likely to bend and deform, thus maintaining its accuracy [31]. In addition, PLA has a higher compression modulus, implying that it is able to withstand higher pressures without deforming. On the other hand, models made of resin do not have the same capacity to withstand pressure, which makes them more susceptible to deformation, the results of this experiment indicate that the material used in PLA are more resistant to external stresses [32]. Gypsum models are known for their inadequate surface hardness and low abrasion resistance, making them highly susceptible to scratches and the deterioration of surface details [33]. In contrast, the surface hardness of PLA-G composite surpasses that of conventional gypsum materials commonly utilized in clinical settings, the addition of different additives to PLA can even affect the accuracy and mechanical properties of PLA products [34, 35]. This improvement suggests that dental models constructed from PLA-G may be wear-resistant and more long-lasting, showing less vulnerability to external forces that could compromise their accuracy compared to traditional gypsum models. In the hardness test conducted on the paper, it was found that the PLA-Gypsum composite material exhibited the highest hardness with a value of 19.48 ± 2.12 Hv1. On the other hand, the resin sample showed the lowest hardness with a value of 10.84 ± 0.50 Hv1. The data of the hardness test implies that the samples produced using the PLA-Gypsum composite material may have greater strength and wear resistance. Although pure PLA exhibited superior performance compared to PLA-G in bending and compression tests during this experiment, it does not necessarily imply that the mechanical properties of PLA are inherently stronger than those of PLA-G. The experimental study conducted by Liao et al. [36] indicated that the porosity of PLA samples significantly influences their mechanical properties, with a decrease in strength correlating with an increase in porosity. Specifically, the porosity levels of the PLA samples in our study were noted to be 1.6% and 4.0% for bars and cylinders, respectively, while the corresponding porosity levels for the PLA-G samples were recorded at 3.0% and 6.0% (bars and cylinders, respectively), the porosity image of PLA-G shown in Fig.6. This disparity in porosity is a crucial factor contributing to the markedly different mechanical properties observed between the two materials, this may also be the reason why PLA-G samples are weaker than pure PLA in this study.

In this research study, we aimed to evaluate the precision and trueness of clinical models produced by 3D printing using three different materials. The experiment yielded results indicating that there was no significant statistical difference in the authenticity of the clinical models produced by the three materials. However, there was a noteworthy statistical difference in the precision of the models produced. The results suggest that the PLA clinical models produced through MEX technology are not significantly different in terms of trueness test when compared to the resin models produced by DLP. Further analysis showed that the dental model made with PLA-G exhibited the highest precision RMS value of 23.84 ± 4.12 μm. On the other hand, the dental model made with resin showed the lowest precision RMS value of 50.74 ± 22.71 μm. Moreover, the study concludes that the clinical models produced with PLA-G are more consistent and exhibit less variation when compared to the two materials, PLA and resin, as demonstrated in Figs. 3 and 4. Overall, these findings provide valuable insights into the use of 3D printing technology for clinical applications and highlight the benefits of using PLA-G for the production of high-accuracy clinical models.

It is worth noting that digital models offer numerous advantages, including ease of use for educational, diagnostic, and therapeutic purposes. Additionally, they provide superior tactile and visual information compared to their traditional counterparts. However, samples produced through 3D printing are not indefinitely available. According to the recommendations of the manufacturer, FFF 3D printing technology does not require post-curing, while models created using DLP technology need to be post-cured to achieve maximum polymerization of the resin with enhanced stability and accuracy [37]. Research by Chen et al. [38] indicated that resin 3D-printed surgical guides lose accuracy after one month of storage. Additionally, experiments conducted by Vanessa et al. [39] highlighted that models made from either PLA or resin materials exhibit significant shrinkage after seven weeks of storage, and models stored in dark conditions showed reduced shrinkage. They concluded that the immediate use of 3D-printed models is preferable.

In recent studies, Lümkemann et al. [40] compared FFF models made from SIMPLEX aligner and Renfert PLA HT filaments with conventional plaster models. The results indicated that the dimensional stability of FFF models, especially with SIMPLEX filaments, was comparable to that of plaster models. Factors such as the number of printing loops and the type of thermoforming foil used were also shown to influence dimensional and stability. These findings highlight the importance of material choice and printing parameters when considering aligner production. We plan to explore similar variables in our next experiment to assess their impact on aligner model’s mechanical stability.

It should be mentioned that only a few studies have established clinically acceptable error ranges for such measurements. A recent study by Yasaman et al. [41] concluded that the majority of scholars currently accept a clinically acceptable error range of <100–500 µm for dental models made using 3D printing technology, furthermore, the study concluded that 3D-printed clinical models are a viable alternative to stone models. It is noteworthy that the acceptable clinical margin of error varies significantly across different dental disciplines. For instance, it seems that below 200 µm error is the clinical dental model acceptable error range [42]. In the other study, in orthodontics, an acceptable clinical model error between orthodontic casts and 3D printed models has been reported to be <300 µm [43]. In the field of prosthodontics, the margin of error that is deemed clinically acceptable for fixed dental prosthesis is considerably narrower, 100–150 µm appears to be a clinically acceptable range [44]. According to the results obtained from the experiment conducted, it can be reasonably inferred that the clinical oral models created using the three materials satisfy the requirements of orthodontic and prosthetic models in terms of accuracy. Furthermore, it has been observed that the models produced using PLA-G exhibit the highest level of precision. The optical microscope images captured during the study have indicated that the surface of the oral models created by the DLP 3D printer is smoother and has less roughness when compared to the surface of the dental model fabricated using the MEX printer. Additionally, the models created using MEX printers exhibit desirable trueness and precision along with better mechanical properties, and the cost of the printer and materials is relatively low, making 3D printing technology models easier to develop in clinical departments. It is also noteworthy that the completely biodegradable and eco-friendly nature of PLA models makes them an attractive alternative to resin models, thus providing more options for clinicians. In light of these advantages, it can be inferred reasonably that PLA models have a significant potential to replace resin models and can offer numerous choices to clinicians working in the field.

## Conclusion

1. According to the findings in this paper, it can be inferred that the experimental evidence presented does not support the null hypothesis. Specifically, the mechanical properties of three distinct materials - namely PLA, PLA-G, and Resin - are not equivalent to the precision of dental models produced via 3D printing.
2. The findings of this research provide evidential support for utilizing 3D-printed dental models in clinics, specifically as orthodontic study models.
3. Based on the findings of the experiment, it can be objectively concluded that there is no significant difference in the trueness of the resin oral models manufactured by the DLP 3D printer as compared to the PLA and PLA-G oral models made by the MEX 3D printer. Furthermore, the precision of dental models with PLA-G material was observed to be superior to the other two materials used in the experiment. The mechanical properties of PLA and PLA-G by 3D printing are stronger than the resin manufactured by DLP.
